# Tumor response assessment on imaging following immunotherapy

**DOI:** 10.3389/fonc.2022.982983

**Published:** 2022-10-25

**Authors:** Antonia M. Berz, Clarisse Dromain, Naïk Vietti-Violi, Sarah Boughdad, Rafael Duran

**Affiliations:** ^1^ Department of Diagnostic and Interventional Radiology, Lausanne University Hospital, Lausanne, Switzerland; ^2^ Department of Radiology, Charité – Universitätsmedizin Berlin, Corporate Member of Freie Universität Berlin and Humboldt-Universität zu Berlin, Berlin, Germany; ^3^ Department of Nuclear Medicine and Molecular Imaging, Lausanne University Hospital, Lausanne, Switzerland

**Keywords:** tumor response, immunotherapy, immune checkpoint inhibitor, pseudoprogression, iRECIST, imRECIST, PERCIMT, iPERCIST

## Abstract

In recent years, various systemic immunotherapies have been developed for cancer treatment, such as monoclonal antibodies (mABs) directed against immune checkpoints (immune checkpoint inhibitors, ICIs), oncolytic viruses, cytokines, cancer vaccines, and adoptive cell transfer. While being estimated to be eligible in 38.5% of patients with metastatic solid or hematological tumors, ICIs, in particular, demonstrate durable disease control across many oncologic diseases (e.g., in melanoma, lung, bladder, renal, head, and neck cancers) and overall survival benefits. Due to their unique mechanisms of action based on T-cell activation, response to immunotherapies is characterized by different patterns, such as progression prior to treatment response (pseudoprogression), hyperprogression, and dissociated responses following treatment. Because these features are not encountered in the Response Evaluation Criteria in Solid Tumors version 1.1 (RECIST 1.1), which is the standard for response assessment in oncology, new criteria were defined for immunotherapies. The most important changes in these new morphologic criteria are, firstly, the requirement for confirmatory imaging examinations in case of progression, and secondly, the appearance of new lesions is not necessarily considered a progressive disease. Until today, five morphologic (immune-related response criteria (irRC), immune-related RECIST (irRECIST), immune RECIST (iRECIST), immune-modified RECIST (imRECIST), and intra-tumoral RECIST (itRECIST)) criteria have been developed to accurately assess changes in target lesion sizes, taking into account the specific response patterns after immunotherapy. In addition to morphologic response criteria, 2-deoxy-2-[^18^F]fluoro-D-glucose positron emission tomography/computed tomography (^18^F-FDG-PET/CT) is a promising option for metabolic response assessment and four metabolic criteria are used (PET/CT Criteria for Early Prediction of Response to Immune Checkpoint Inhibitor Therapy (PECRIT), PET Response Evaluation Criteria for Immunotherapy (PERCIMT), immunotherapy-modified PET Response Criteria in Solid Tumors (imPERCIST5), and immune PERCIST (iPERCIST)). Besides, there is evidence that parameters on ^18^F-FDG-PET/CT, such as the standardized uptake value (SUV)max and several radiotracers, e.g., directed against PD-L1, may be potential imaging biomarkers of response. Moreover, the emerge of human intratumoral immunotherapy (HIT-IT), characterized by the direct injection of immunostimulatory agents into a tumor lesion, has given new importance to imaging assessment. This article reviews the specific imaging patterns of tumor response and progression and available imaging response criteria following immunotherapy.

## 1 Introduction

In recent years, the success of systemic immunotherapies for cancer treatment has led to a paradigm shift in the field of oncology and has generated great interest in the medical community. Mechanistically, all immunotherapeutic approaches have in common that they target key mechanisms of the tumor microenvironment (TME). Particularly important in this context is the overexpression of immunosuppressive immune checkpoints such as cytotoxic T lymphocyte-associated protein-4 (CTLA-4), programmed cell death protein-1 (PD-1), and programmed death-ligand 1 (PD-L1) in the local TME that may prevent the immune system, in particular T cells, from targeting and destroying cancer cells (1). In 2011, the Food and Drug Administration approved ipilimumab, a monoclonal antibody (mAb) targeting CTLA-4, as the first immune checkpoint inhibitor (ICI) for unresectable metastatic melanoma (2). Today, a broad spectrum of immunotherapeutic agents with various mechanisms of action is available. These agents received marketing authorization for melanoma, lung, bladder, renal, and head and neck cancers and it is estimated that 38.5% of oncological patients can be treated with them (3). All these therapies have in common that they target dysregulated immunologic pathways to break the cancer tolerance and stimulate the antitumor immune response (1, 4–7). ICIs in particular showed durable disease control across many oncologic diseases and overall survival (OS) benefits (1). However, all these expensive therapies are limited due to the relatively small number of patients achieving an objective response, various systemic immunotherapy-related adverse events (irAEs), and long-term therapy resistance (8–11).

Treatment response to immunotherapies is characterized by different patterns, such as tumor progression prior to response (pseudoprogression), hyperprogression following treatment, and mixed/dissociated responses (12). These patterns are often observed in patients treated with immunotherapies, in particular ICIs, although some of these patterns (e.g. dissociated response) may also be seen following chemotherapy and targeted therapies (13). The Response Evaluation Criteria in Solid Tumors version 1.1 (RECIST 1.1) is the standard of care for evaluating changes in tumor size to assess treatment response following systemic therapies in a quantitative and presumably objective manner (14). Because these criteria proved to be inadequate in the setting of immunotherapy they have been fully modified and repeatedly adapted to accurately assess response after different immunotherapeutic approaches (15–20). However, all these assessment methods are complex and often complicated to apply in clinical practice.

Recently, 2-deoxy-2-[^18^F]fluoro-D-glucose positron emission tomography/computed tomography (^18^F-FDG-PET/CT) has demonstrated its potential in the context of immunotherapy for characterizing these response patterns, assessing treatment responses using metabolic response criteria, imaging irAE and even providing information about patient prognosis, mainly in patients with non-small cell lung cancers and advanced melanomas (21, 22).

Human intratumoral immunotherapy (HIT-IT), which is characterized by the direct injection of immunostimulatory agents into a tumor lesion (primary or metastatic), is a potential promising option to overcome many immunotherapy-related problems, such as immune tolerance (23). Direct exposure of the tumor cells to immunotherapeutic agents leads to stronger local immune responses in the injected (“enestic”) lesion while requiring smaller amounts of the drugs per patient, causing fewer systemic side effects and off-target toxicities (24). The tumor is therefore used as its own vaccine by eliciting polyclonal B- and T-cell mediated adaptive immune responses against pre-existing tumor-specific and tumor-associated antigens, that produce abscopal effects in distant, non-injected (“anenestic”) tumor sites (23). For instance, as the first HIT-IT for IIIb-IVM1a melanoma, the herpes-derived genetically modified oncolytic virus called Talimogene laherparepvec (T-VEC) was recently approved by the Food and Drug Administration/European Medicines Agency (25–27). In addition, there is (pre)clinical evidence that intratumoral injections of mAbs directed against CTLA-4 and/or PD(L)-1 might enable to overcome resistance to systemic ICIs by inducing an effective intratumoral T-effector cell homing (28–31). Many other drugs are currently under investigation to treat several solid tumors (27). Anticipating the increased use of HIT-IT, specific imaging criteria, the intratumoral RECIST (itRECIST), have been developed for response assessment.

This article aims to review the specific imaging patterns of response and progression as well as available imaging response criteria following immunotherapy.

## 2 Response assessment

### 2.1 Characteristics of response and progression

Specific (radiologic) patterns of treatment response are frequently observed in patients treated with immunotherapies and are related to their unique indirect mechanism of action targeting the immune system rather than the tumor cells (13).

#### 2.1.1 Onset of response and durable responses

**Figure 1:**
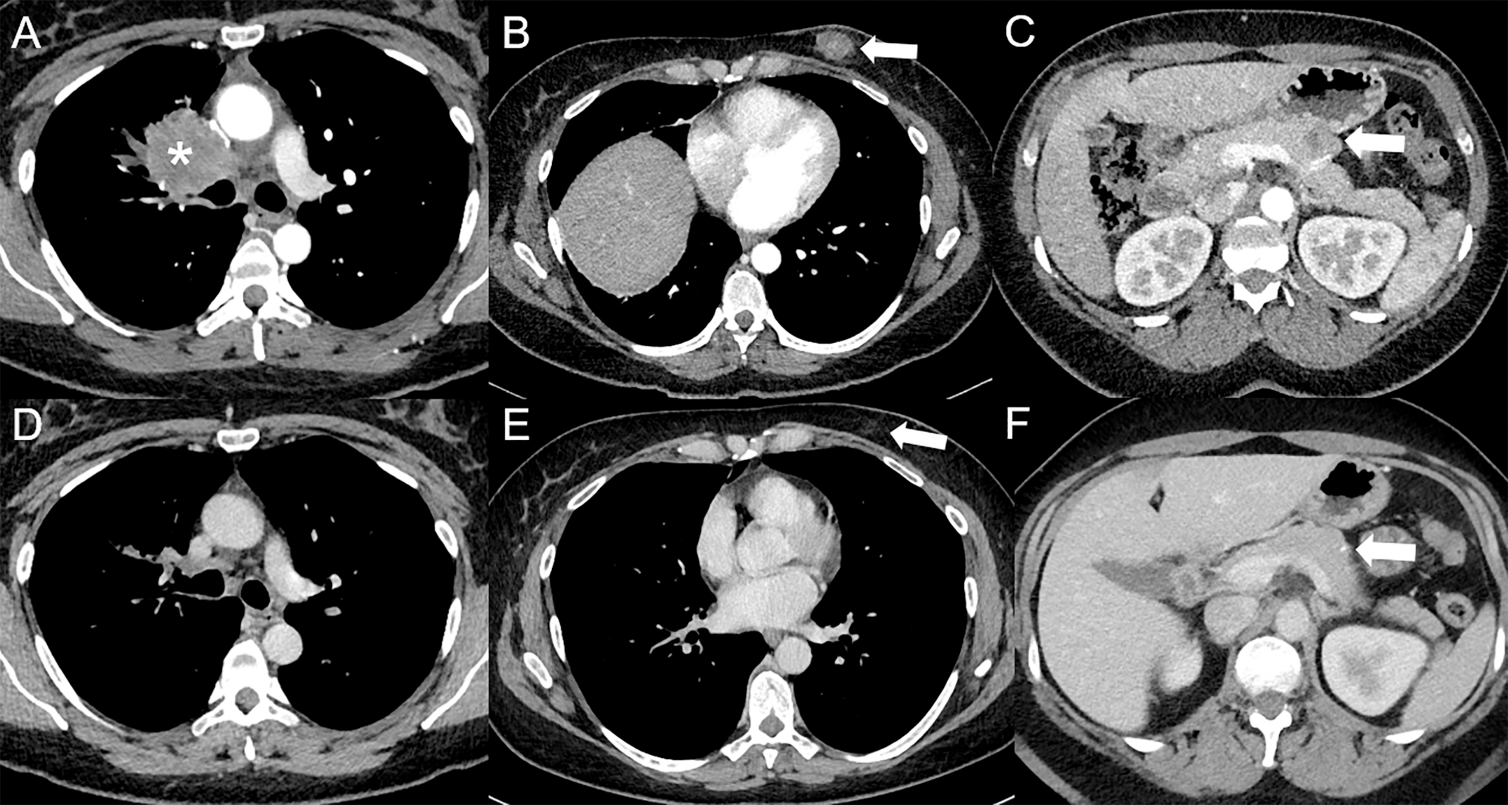
Complete response following immunotherapy in a 41-year-old female diagnosed in February 2016 with stage IV poorly differentiated lung adenocarcinoma in right upper lobe [asterisk *, **(A)**], with hypodense subcutaneous (arrow, **(B)**), and pancreatic [arrow, **(C)**] metastases visible on baseline contrast-enhanced CT at the arterial phase **(A–C)**, treated with durvalumab (anti-PD-L1) and tremelimumab (anti-CTLA-4), followed by durvalumab maintenance. Since May 2017, a complete response was obtained. In April 2022, a complete response was still observed at the primary tumor **(D)** and subcutaneous [arrow, **(E)**] sites, whereas only a squealer calcification [arrow, **(F)**] can be seen at the pancreatic site on contrast-enhanced CT at the portal venous phase **(D–F)**.

Classical chemotherapy reduces tumor-growth kinetics mainly during administration and regrowth of the lesions is observed following treatment discontinuation. Similarly, targeted therapies, that block driver oncogenes, have shown to induce a rapid tumor response that is, however, usually not durable (32). In contrast, immunotherapeutic strategies have the notorious ability to elicit delayed but durable responses, as they stimulate cancer-specific T-cell mediated immune infiltration, albeit responses are observed in only 10-20% of the patients (**Figure 1**) (17, 32, 33). As a breakthrough in melanoma therapy, these treatments have induced long-lasting remission of more than five years (34). A recent analysis of a subset of melanoma patients treated with ICIs who survived at least 5 years (n=151) demonstrated a median duration of response of 93 months among survivors, only 4 patients experienced disease progression after 5 years, and none of them ultimately died from melanoma (35). A meta-analysis including 19 studies showed a proportion of durable response in 25% of the patients treated with ICIs, which is 2.3 times higher than of those treated without ICIs (11%). Durable responses were more frequent in patients treated with anti-PD-1/PD-L1 drugs than in patients treated with anti-CTLA-4 agents (36). Being persistent even after treatment discontinuation, durable responses question the common concept of continuing the treatment until disease progression. Interestingly, the median onset of response after HIT-IT with T-VEC was 3.1 months, which is even later than after treatment with ICIs such as nivolumab (anti-PD1 mAb), where the median time to response takes 2.2 months; both time points were obtained in phase III clinical trials in melanoma patients (33, 37, 38). Thus, the itRECIST suggest 4 to 12 weeks rather than 4 to 8 weeks for the immune RECIST (iRECIST) for appropriate response assessment (cf. chapter “criteria of tumor response assessment”) (16). Consequently, the time point for response assessment of HIT-IT is chosen later in order to allow sufficient time for the treatment to exert its effect. Recommended assessment time points for each response criteria are summarized in **Table 1**.

**Table 1:** Morphologic criteria for the assessment of response to immunotherapy.

	Lesion definition	CR	PR	SD	PD	Confirmation of PD	New lesions
**RECIST 1.1** (14), 2009	Uni-dimensional ≥ 10mm, 5 lesions, 2/organ	Disappearance of all lesions	≥ 30% decrease form baseline	Neither CR nor PD	≥ 20% increase from the nadir (≥ 5mm)	Not applicable	PD
**irRC** (17), 2009	Bi-dimensional, 5x5 mm 15 lesions, 5/organ	Disappearance of all lesions	≥ 50% decrease form baseline	Neither CR nor PD	≥ 25% increase from the nadir	At least 4 weeks	Incorporated to the sum of measurements
**irRECIST** (18), 2013	Uni-dimensional, ≥ 10mm, 5 lesions, 2/organ	Disappearance of all lesions	≥ 30% decrease form baseline	Neither CR nor PD	≥ 20% increase from the nadir (≥ 5mm)	4-12 weeks	Incorporated to the sum of measurements
**iRECIST** (19), 2017	Uni-dimensional, ≥ 10mm, 5 lesions, 2/organ	Disappearance of all lesions	≥ 30% decrease form baseline	Neither CR nor PD	≥ 20% increase from the nadir (≥ 5mm)	4-8 weeks	iuPD
**imRECIST** (20), 2018	Uni-dimensional, ≥ 10mm, 5 lesions, 2/organ	Disappearance of all lesions	≥ 30% decrease form baseline	Neither CR nor PD	≥ 20% increase from the nadir (≥ 5mm)	At least 4 weeks	Incorporated to the sum of measurements
**itRECIST** (16), 2020	Uni-dimensional, ≥ 10mm, 10 lesions (5 injected, 5 not injected)	Disappearance of all lesions	≥ 30% decrease form last exam for injected lesions, ≥ 30% decrease form baseline for not injected lesions	Neither CR nor PD	≥ 20% increase from the nadir (≥ 5mm)	4-12 weeks	iuPD

CR, complete response; PR, partial response, SD, stable disease; PD, progressive disease; iuPD, unconfirmed progressive disease; RECIST 1.1, Response Evaluation Criteria in Solid Tumors version 1.1; irRC, immune-related response criteria; irRECIST, immune-related RECIST; iRECIST, immune RECIST; imRECIST, immune-modified RECIST; itRECIST, intra-tumoral RECIST.

#### 2.1.2 Pseudoprogression

**Figure 2:**
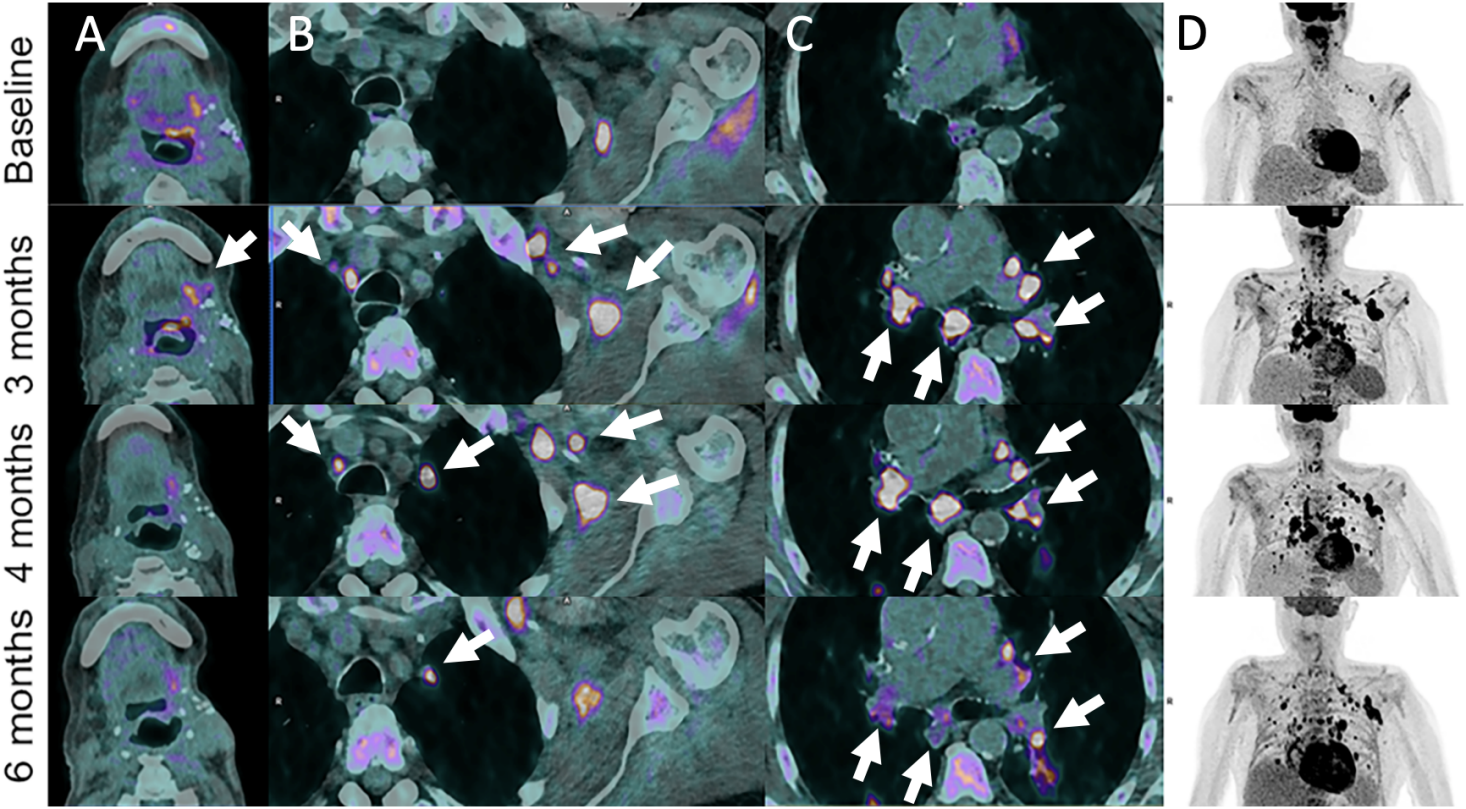
Pseudoprogression in 72-year-old male patient with left base of tongue squamous cell carcinoma recurrence after initial surgery with satellite cervical, axilla and mediastinal lymphadenopathies as shown on ^18^F-FDG-PET/CT images (Baseline, **A–D**). Pembrolizumab (anti-PD-1) was administered. Follow-up imaging at 3 months post-pembrolizumab administration shows local increase in metabolism together with hypermetabolic left axillar and symmetrical mediastinal and hilar lymph node enlargement (sarcoidosis-like reaction) (arrows). These features progressively disappeared or markedly improved at 4- and 6-months follow-up.

Progression prior to response, known as pseudoprogression, is defined as an initial increase of the tumor burden (including new lesions) that is not confirmed at the next imaging follow-up (**Figure 2**) (39). There are two main biological reasonings to explain pseudoprogression in the setting of immunotherapy. The first one is the time required to mount an adaptive immune response due to the indirect mechanism of action of immunotherapy, during which tumor growth continues (40). The second explanation, which has been confirmed by histopathological analysis of tumor specimens, is a transient local lymphocytic infiltration into the tumor with cytokine production leading to the radiologic image of an increased tumor size (41). First described in a phase II trial that evaluated the efficacy of ipilimumab in advanced melanoma, the rate of pseudoprogression following ICIs ranged from 1.3% to 9.1% and never exceeded 10% of patients, depending on the tumor type and administered drug (17, 39). This is much lower than the rate of true progression. Thus, an increase of the tumor burden during ICI treatment is more likely to actually reflect true progression than a pseudoprogression. Interestingly, pseudoprogression has been reported to be more frequently observed after HIT-IT, occurring in 48% of patients treated with intratumoral injections T-VEC, who show later a durable response (37). Being not a true proliferation associated with progressive disease (PD), pseudoprogression even underscores the importance of continued treatment (17, 37, 42).

**Figure 3:**
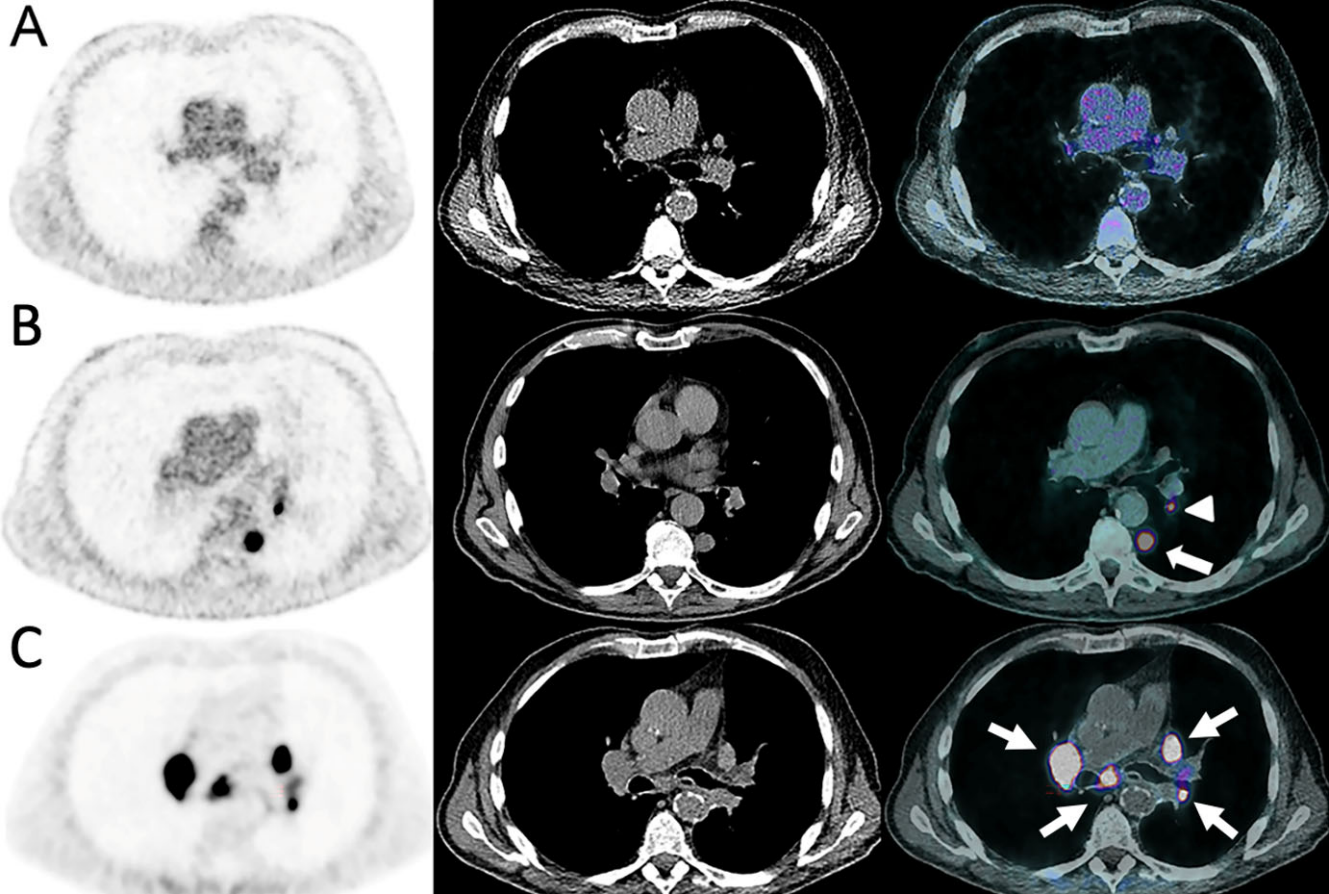
Sarcoidosis-like reaction in an 84-year-old male with cutaneous melanoma of unknown primary. The disease initially presented as the incidental discovery of a lung nodule on CT **(A, B)**. A^18^F-FDG-PET/CT was done and showed a solitary left pulmonary lower lobe nodule (arrow) with a homolateral hilar lymph node (arrowhead) which were both highly metabolic. Mediastinum and upper hilum were disease free. The patient underwent upfront surgery which established the diagnosis. Nivolumab was administered 1 month post-surgery and after 4 cycles the patient developed dyspnea. **(C)** A^68^Ga-DOTATOC-PET/CT showed intense and diffuse uptake in enlarged mediastino-hilar lymph nodes (arrows) suggesting a reactive inflammatory process with high uptake of activated lymphocytes strongly expressing STTR-2 targeted by ^68^Ga-DOTATOC.

Another differential diagnosis to consider when new lesions appear during ICI treatment is immune-related side effects. A commonly tricky radiological irAE, that should not be misinterpreted, are sarcoid-like reactions. Sarcoid-like reactions manifest as new bilateral symmetric mediastinal and hilar lymphadenopathy, which may appear hypermetabolic on ^18^F-FDG-PET/CT (**Figure 3**) (43, 44). The advent of concomitant organizing pneumonia-like pneumonitis is also typical and suggestive when new lung nodules demonstrate a reversed halo sign or in case of the appearance of confluent consolidations with or without air bronchograms which are predominant in a peripheral or subpleural distribution (43, 45). Diffuse lymph node enlargement and adrenalitis with uni- or bilateral adrenal gland enlargement and mild ^18^F-FDG avidity on PET/CT can also be observed (10, 11).

The presence of inflammatory effects due to the activation of the immune system and subsequent intratumoral lymphocyte infiltration during the course of immunotherapy could affect the assessment of tumor response using ^18^F-FDG-PET/CT as it is well known that inflammatory findings often present high glycolytic activity. In addition, the upregulation of glucose transporter mRNA and proteins in the TME resulting from anti-PD-1 activation in patients treated with ICIs can lead to increased ^18^F-FDG uptake (46). Therefore, they might be reported as pseudoprogression (17, 47). In a population of non-small cell lung cancer patients, more than 50% of the patients with PD according to the PET Response Criteria in Solid Tumors (PERCIST) on interim PET/CT (7 weeks after initiation of anti-PD1 mAb treatment with pembrolizumab or nivolumab) that continued the ICI treatment had a response or stable disease (SD) on the following PET/CT, defining pseudoprogression (48).

#### 2.1.3 Hyperprogression

**Figure 4:**
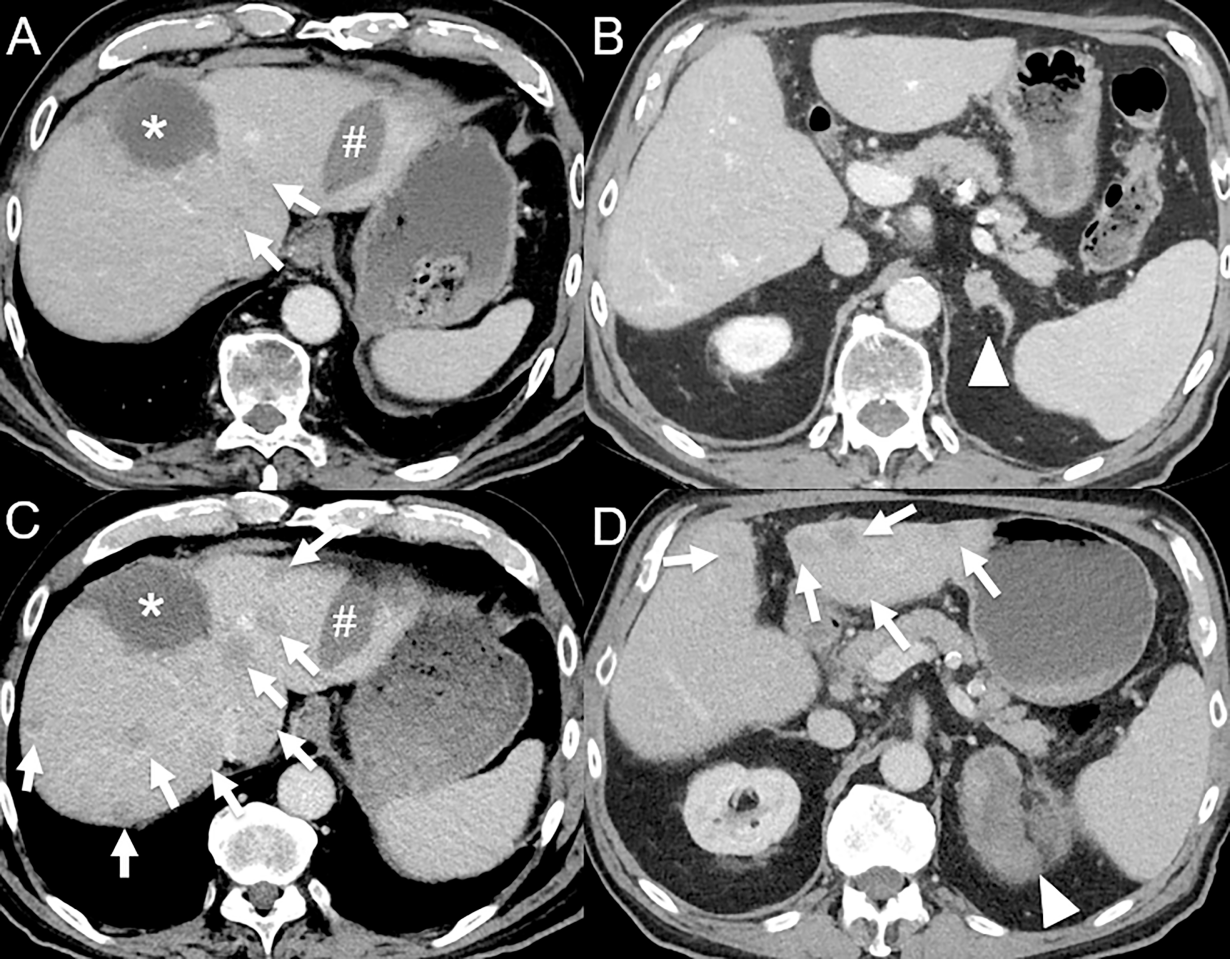
Hyperprogression in an 82-year-old male with hepatocellular carcinoma initially treated by selective internal radiation therapy and radiofrequency ablation who develop metastatic disease 11 months post-therapy (bone, left adrenal gland and lung). **(A)** Baseline contrast-enhanced CT at the portal venous phase shows necrotic lesion treated by selective internal radiation therapy (*) and a radiofrequency ablation scar (#), with 2 small hypodense lesions (arrows). **(B)** A left adrenal gland metastasis is also seen (arrowhead). The patient was given atezolizumab (anti-PD-L1) and bevacizumab (anti-VEGF). 1-month follow-up contrast-enhanced CT at the portal venous phase shows dramatic disease progression with the appearance of multiple cancer lesions [**(C, D)**, arrows] and marked increase in the left adrenal gland metastasis [**(D)**, arrowhead].

**Figure 5:**
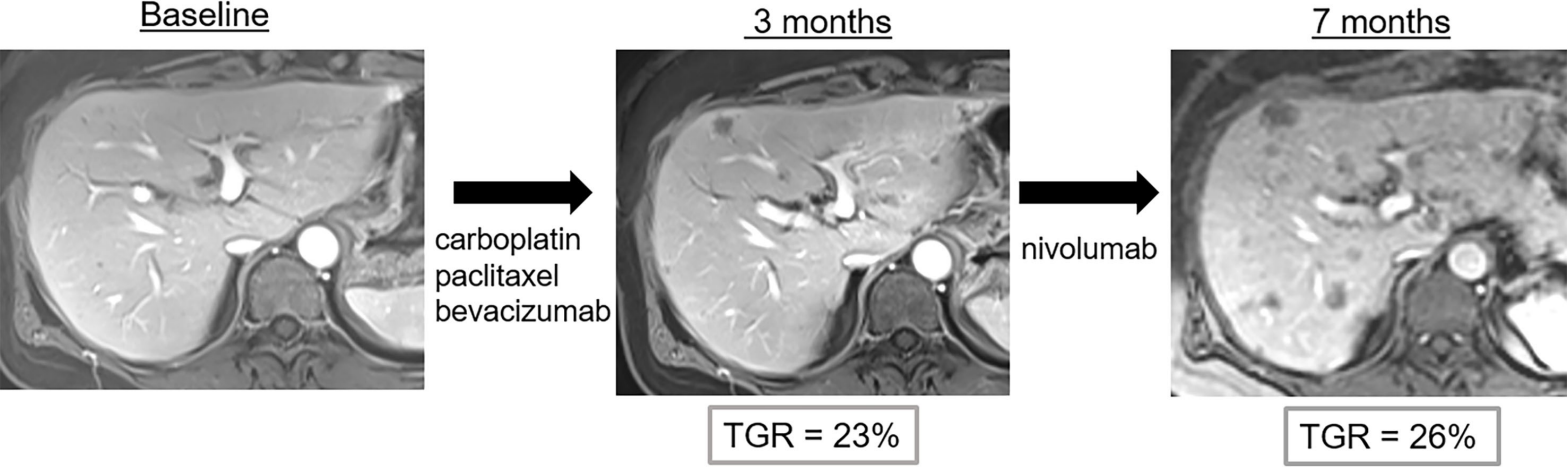
Pseudo-hyperprogression in a 53-year-old female with anal squamous cell cancer who showed disease progression following treatment with carboplatin, paclitaxel and bevacizumab. Nivolumab (anti-PD1) was then administered. Imaging follow-up showed continuous progression of liver metastases with potential hyperprogression after initiation of immune checkpoint blockade therapy. However, the tumor growth rate (TGR) did not significantly change when compared to baseline imaging. Thus, the diagnosis of hyperprogression was wrong and imaging findings correspond to disease evolution.

Hyperprogression, a well-known pattern of response after immune checkpoint blockade, is characterized by the paradoxical acceleration of tumor growth kinetics after initiation of immunotherapy (**Figure 4**) (49). There are several definitions of hyperprogression, all of which can be described as RECIST progression at the initial imaging assessment, but it is most commonly defined as an ≥ 2-fold increase in tumor growth rate (TGR) (50, 51). The ultimate biological rationale underlining hyperprogression remains unknown. However, there is evidence that older age, amplification of the double minute 2 homolog gene, epidermal growth factor receptor alterations, the tumor mutational burden, and TME modification (e.g. by previous ablation or radiotherapy) may be critical mechanisms for this event (50, 51). While being reported in 4% to 29% of patients across retrospective studies, hyperprogression is associated with poor prognosis and survival outcomes (52). Of note, hyperprogression is a major subject of debate in the medical community. Considering the limited number of reported cases of hyperprogression compared with the number of patients receiving immunotherapy, the fact that all these studies were retrospective with no control arm, and the heterogenous definition of hyperprogression, some physicians still consider that this could reflect the natural history of the disease. In addition, there are cases of pseudo-hyperprogression which initially showed signs of hyperprogression, but the TGR never changed significantly when compared to baseline imaging (**Figure 5**). For ^18^F-FDG-PET/CT, there are still no robust evidence based parameters that could predict hyperprogression. However, it has been reported that melanoma patients who presented hyperprogression had significantly higher baseline metabolic tumor volume (MTV, metabolically active volume of the segmented tumor), total lesion glycolysis (TLG, product of MTV and mean standardized uptake value (SUVmean)) and total measured tumor volume burden (53). Similarly, an increased risk for hyperprogression was found in non-small cell lung cancer patients treated with ICIs with higher MTV and an increased derive neutrophil-to-lymphocyte ratio, suggesting that the definition of a multiparameter model could improve the prediction of tumor response (54).

#### 2.1.4 Dissociated response

**Figure 6:**
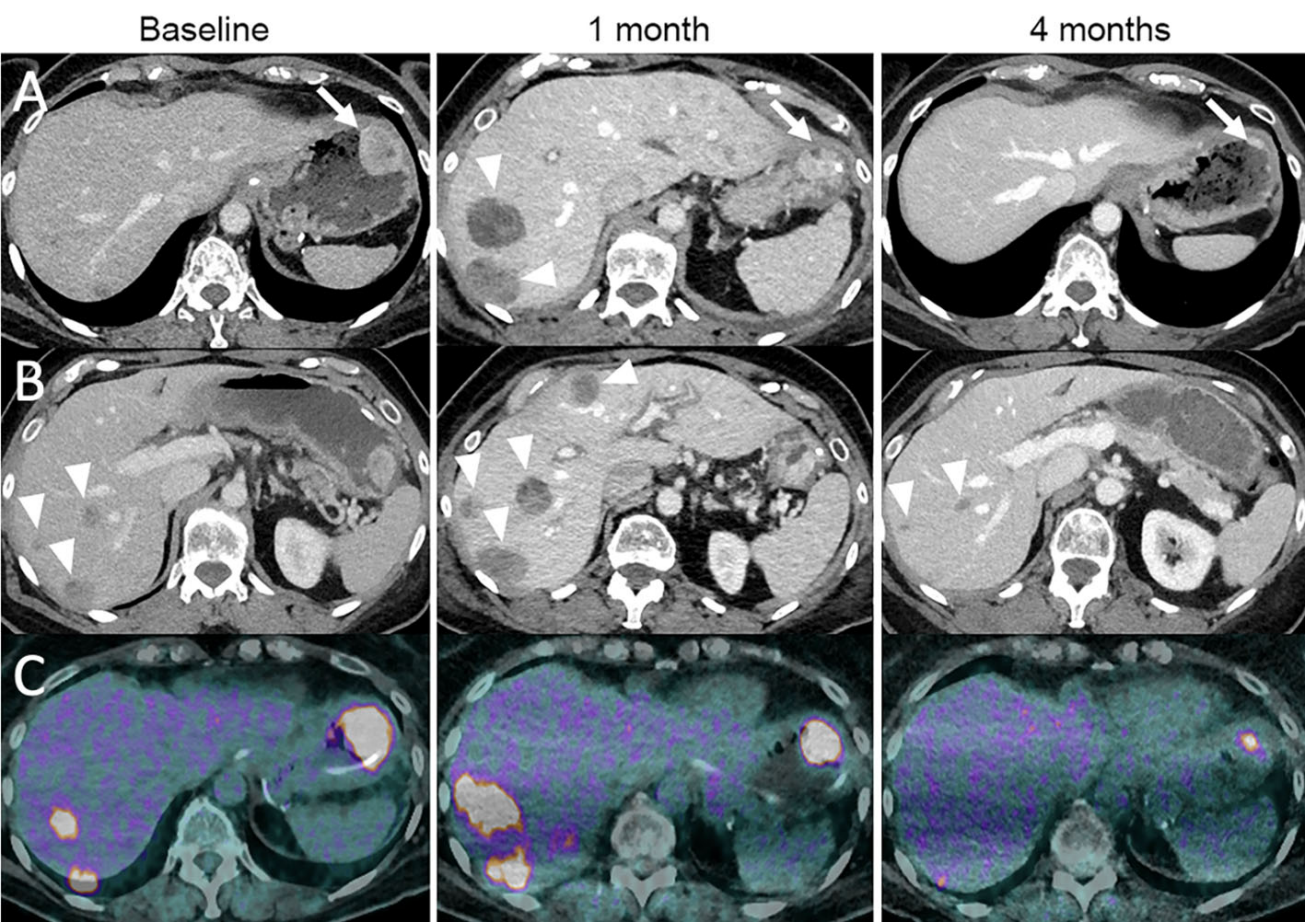
Dissociated response in a 68-year-old female with cutaneous melanoma of the back who progressed following ipilimumab (anti-CTLA-4) and nivolumab (anti-PD-1). The patient was given tumor-infiltrating lymphocyte-adoptive cell therapy (TIL-ACT). Baseline contrast-enhanced CT at the portal venous phase **(A, B)** and ^18^F-FDG-PET/CT **(C)** show a gastric wall metastasis (arrow) and liver metastases (arrowheads). 1-month follow-up imaging after TIL-ACT demonstrates a decrease in the gastric wall metastasis (arrow). However, liver metastases increased in size (arrowheads). 4-month follow-up imaging shows a marked decrease in the gastric wall metastasis (arrow) and liver metastases (arrowheads).

**Figure 7:**
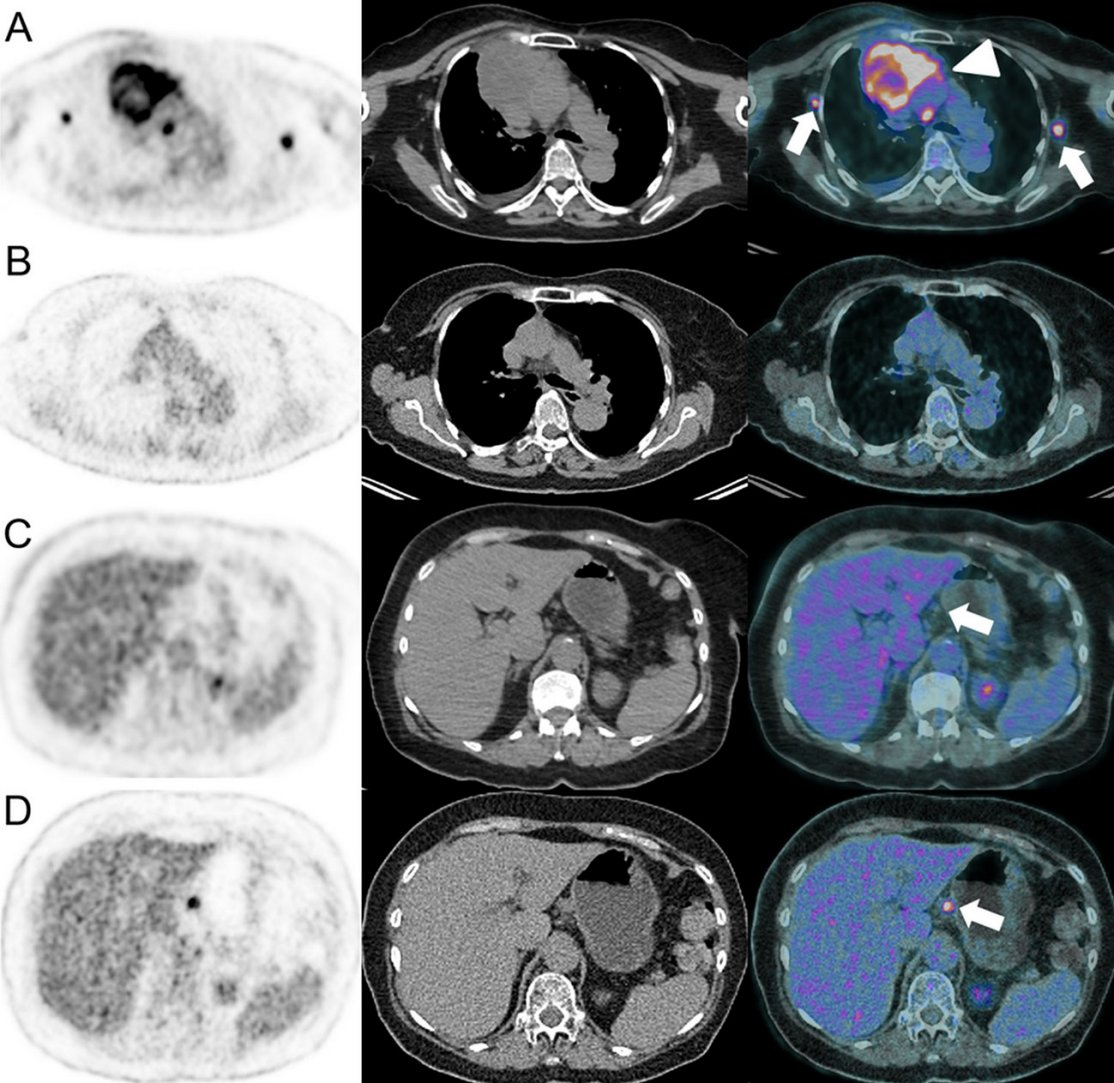
Dissociated response in a 63-year-old female with left lower leg Meckel carcinoma and mediastinal [**(A)**, arrowhead] and axillar [**(A)**, arrows] metastatic spread as demonstrated on baseline ^18^F-FDG-PET/CT **(A)**. Pembrolizumab (anti-PD-1) was administered. At the 6-months follow-up, ^18^F-FDG-PET/CT showed complete response on the mediastinal and axillar lymph nodes **(B)**. However, a hypermetabolic coeliac lymph node appeared [**(D)**, arrow], which was not present at baseline [**(C)**, arrow].

On morphological imaging, dissociated response, also called mixed response, is defined as the coexistence of responding [complete response (CR) or partial response, (PR)] and non-responding (SD or PD) lesions according to RECIST 1.1 within the same patient (**Figure 6**) (55, 56). In ^18^F-FDG-PET/CT studies, the term immune-dissociated response (iDR) was defined as decrease in some hypermetabolic lesions associated with an increase in other lesions, in contrast to immune homogeneous PD (**Figure 7**) (48). Dissociated responses have been reported in 3.3% to 9.2% of patients treated with ICIs and show better OS than true progression (13, 57–59). Several physiopathological hypotheses may explain this phenomenon, such as genomic tumor heterogeneity and differences in the TME between the distinct metastatic sites (55, 60).

Although there is evidence that dissociated responses are associated with treatment efficacy, such as in 10% of ICI-treated advanced lung cancer patients, current response criteria (RECIST1.1 and iRECIST) often (mis-)classify this pattern as PD (48, 55). Apart from this, radiologists should consider some pitfalls such as synchronous cancers, treatment-related side effects (e.g., sarcoïdose-like reaction), specific response patterns (e.g., pseudoprogression of a single metastatic site), and inflammation-induced tracer uptake on ^18^F-FDG-PET/CT in the differential diagnosis of dissociated response. However, no association could be found between dissociated response and the site of metastases in cancer patients treated with ICIs, but in general, liver metastases were less responsive to immunotherapy than lung metastases and metastatic lymph nodes (56). Importantly, dissociated reponse have to be clearly mentioned in the radiology report to evaluate the possibility of a local treatment in cases of oligometastatic PD.

Of note, this pattern is particularly challenging when evaluating the response to HIT-IT. Mixed responses following HIT-IT occur when injected lesions disappear, but new tumor foci developed, or the size of primarily not-injected lesions increase simultaneously. Interestingly, a phase III trial of stages IIIB–IV melanoma patients treated with T-VEC showed that the response rate, defined as decrease of the lesion size ≥50 %, was 64 % in injected lesions, 34 % in not-injected non-visceral lesions, and 15 % in not-injected visceral lesions (61). Complete resolution has been shown by the same study to occur in 47 % of injected lesions, in 22 % of not-injected non-visceral lesions, and in 9 % of not-injected visceral lesions. In these cases, physicians must reevaluate which lesions to inject, carefully weighing the benefits of therapy against the patient’s own risk factors and potential treatment-related complications (16).

### 2.2 Criteria of tumor response assessment

#### 2.2.1 Morphologic criteria

Specific morphologic criteria for patient response evaluation to immunotherapy are summarized in **Table 1**. These criteria are mainly an adaptation of the traditional RECIST 1.1 (14) and World Health Organization criteria (62). However, they have two major differences:

1. the need for a confirmatory imaging examination in case of lesion progression and
2. the appearance of new lesions is not necessarily considered as progression criterion.

##### 2.2.1.1 irRC

The immune-related response criteria (irRC) were first elaborated for melanoma patients treated with ipilimumab (17). They are based on the World Health Organization criteria (bi-dimensional tumor measurements, 5 lesions per organ with up to 10 visceral lesions and 5 cutaneous index lesions) (62). In the irRC, CR is defined as complete disappearance of all target lesions, and PR is defined as reduction of ≥ 50% of the sum of target lesions compared with the baseline. PD is defined as ≥ 25% increase of the sum of target lesions compared with the nadir and/or appearance of new measurable lesions (tumor lesions ≥ 10mm measured in the long axis and lymph nodes ≥ 15 mm measured in the small axis). SD is considered when the response does not qualify for PR nor PD (**Table 1**).

##### 2.2.1.2 irRECIST

The immune-related RECIST (irRECIST), were developed based on the RECIST 1.1 and use unidimensional measurements (18). The irRECIST is more adapted for clinical practice and has shown to provide a highly concordant response assessment with low measurement variability and, therefore, a higher reproducibility compared with the irRC (18). In addition, it has the advantage of enabling direct comparison with RECIST 1.1, thus allowing for comparison within clinical trials. The irRECIST defines PR as a reduction of ≥ 30% of the sum of the target lesions compared with the baseline, PD as a > 20% increase of the sum of the target lesions compared with the nadir and/or new measurable lesions. SD is defined as an increase of < 20% and a decrease < 30% of the sum of the target lesions compared with the baseline (**Table 1**).

##### 2.2.1.3 iRECIST

In 2017, the RECIST working group proposed another modified version of the RECIST 1.1 for immune-based therapies, the iRECIST (19). The definitions of iPR, iSD and iPD are the same as in the irRECIST (**Table 1**). However, the iRECIST introduced the new concept of “unconfirmed progressive disease” (iuPD). Briefly, iuPD corresponds to PD (sum of the tumor lesions increase by ≥ 20% compared with the nadir, non-target lesions progress, new lesions occur) which is not confirmed at the next imaging session within 4 to 8 weeks (**Table 1**). If an iuPD can be documented, the treatment should be continued.

In contrast to the irRECIST, in the iRECIST, the definition of a confirmed PD (icPD) is precisely determined by the following conditions at the 4 to 8 weeks imaging follow-up after iuPD:

1. a further increase of the lesions (≥ 5mm additional increase),
2. a significant increase of a non-target lesion previously classified as iuPD,
3. an increase in the size (≥ 5mm) of a previously new lesion, or
4. the appearance of new lesions.

**Figure 8:**
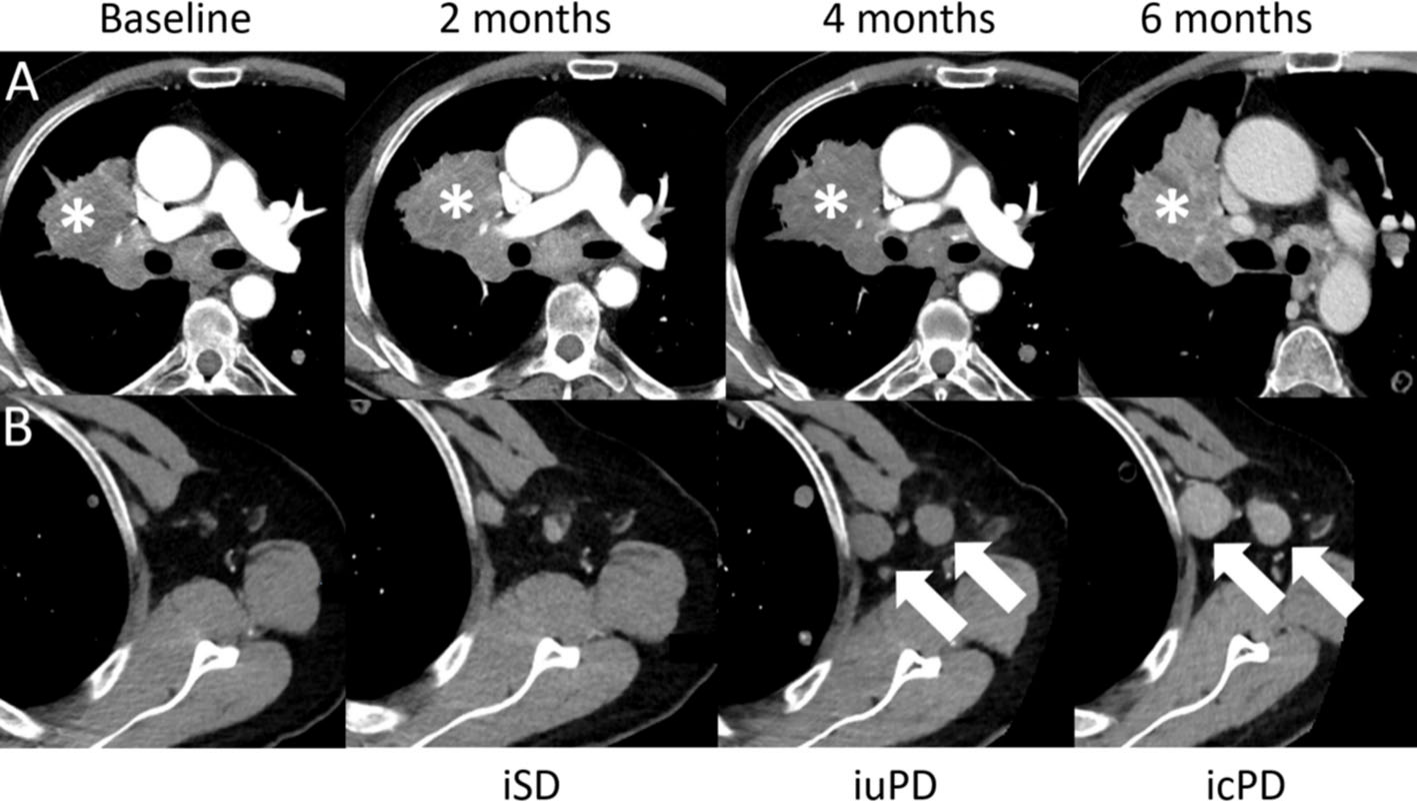
Confirmed progressive disease (icPD) in a 64-year-old male with right hilum adenocarcinoma treated with nivolumab (anti-PD-1) and anti-Lymphocyte Activation Gene-3 (LAG-3). Baseline contrast-enhanced CT demonstrates the right hilum mass (**A**,*). The 2-months follow-up showed stable disease (iSD). However, at the 4-months follow-up, the right hilum mass grew (**A**,*) and enlarged axillar lymph nodes appeared [**(B)**, arrows], consistent with unconfirmed PD (iuPD). At the 6-months follow-up, confirmed PD (icPD) was established with continued growth of the right hilum mass (**A**,*) and the axillar lymph nodes [**(B)**, arrows].

If icPD is confirmed, the first date of iuPD is the event date for the progression-free survival (PFS) assessment (**Figure 8**).

##### 2.2.1.4 imRECIST

The immune-modified RECIST (imRECIST) were initially developed for atezolizumab (anti-PD-L1 mAb) clinical trials of non-small cell lung cancer, melanoma, metastatic urothelial and renal cell carcinomas (20). Except for the use of unidimensional measurements and a modification of the PFS assessment, these criteria are very similar to the irRC. In the imPFS assessment, PD or death is still considered as an event. However, if the follow-up scan (≥ 4 weeks) shows SD, PR, or CR, the initial PD is not considered an imPFS event. If there is no subsequent imaging assessment, PD is considered as imPFS event. Similar to irRC, new measurable lesions are incorporated into the sum of the target lesions. Moreover, an increase of ≥ 20% of the sum of target lesions from the nadir as well as new measurable lesions are considered as PD, which should be confirmed at a ≥ 4 weeks imaging follow-up (**Table 1**).

##### 2.2.1.5 itRECIST

**Figure 9:**
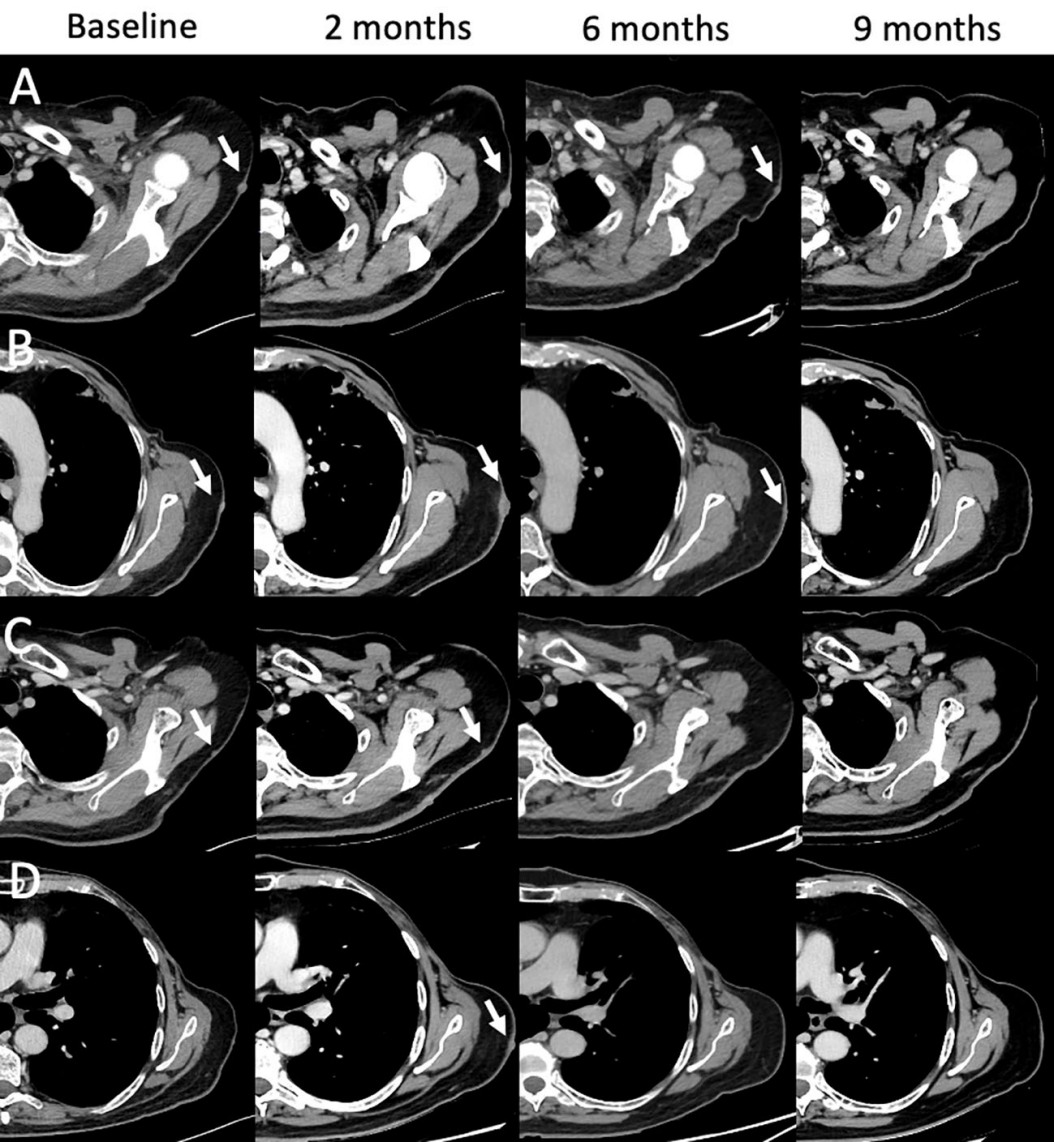
Mixed and complete responses following T-VEC therapy in a 66-year-old male with stage IIIB nodular multilesional melanoma in the left arm (baseline contrast-enhanced CT, arrows) previously treated with surgery and systemic immunotherapy with ipilimumab & nivolumab. Treatment with T-VEC was performed in up to 8 subcutaneous lesions per session. Following 3-4 treatment cycles, follow-up CTs were performed (at 2, 6, and 9 month). Representative examples of lesion evolution since baseline **(A–D)**: Follow-up at 2 months showed mixed responses with progressive lesions [**(A, B)**, arrows], stable lesions [**(C)**, arrow] and new lesions [**(D)**, arrow]. Follow-up at 4 months showed partial responses **(A, B)** and complete responses **(C, D)**. Follow-up at 9 months showed complete response in all lesions **(A–D)**. Treatment with T-VEC was continued.

With the introduction of HIT-IT, the intra-tumoral RECIST (itRECIST) were developed (16). Response assessment principles are comparable to iRECIST with the particularity that 5 injected lesions and 5 not-injected lesions are evaluated separately. Because injected lesions may change within the treatment cycles, these criteria always compare injected lesions to both the size of the target lesions at the last imaging examination and to the baseline or nadir, as in the assessment of not-injected lesions (**Figure 9**). If a PD has been defined, a confirmatory imaging follow-up is required after 4 to 12 weeks (**Table 1**).

#### 2.2.2 Metabolic criteria

In 1999, the European Organization for Research and Treatment of Cancer’ (EORTC) introduced the first metabolic ^18^F-FDG-PET/CT based assessment criteria for oncological disease evaluation (63). Several years later, these EORTC criteria were superseded by PERCIST (64). These new criteria introduced the concept of the SUV normalized by the lean body mass (SUL). A tumor SUL 1.5-fold higher than the SUL of the non-affected liver has been defined as prerequisite for an evaluable lesion. SUL_peak_ is assessed within a spherical volume of interest in the most metabolic active tumor region. In a small patient population with non-small cell lung cancer, the presence of a tumor response according to PERCIST and EORTC criteria on early ^18^F-FDG-PET/CT after 2 or 3 immunotherapy cycles was associated with CR/PR and could even predict post-treatment progression (63–65). However, the limitations of PERCIST and EORTC to accurately assess tumor response in patients treated with immunotherapy have led to the suggestion of modified response assessment criteria that were initially developed in patients with advanced melanoma undergoing ICI therapy (66, 67). These specific metabolic criteria to assess therapy response to immunotherapy are summarized in **Tables 2**, **3**.

**Table 2:** Metabolic criteria for the assessment of response to immunotherapy.

	Lesion definition	CMR	PMR	SMD	PMD	Confirmation of PD	New lesions
**EORTC** (63), 1999	SUVmean, normalized body surface (no prespecified number of target lesions)	Complete resolution of FDG uptake within tumor volume so that it is indistinguishable from surrounding normal tissue	Reduction of 15–25% in tumor SUV after 1 cycle of therapy and > 25% after more than 1 cycle of therapy	Increase in tumor SUV of < 25% or decrease of < 15% and no visible increase in extent of ^18^F-FDG tumor uptake (20% in longest dimension)	Increase from baseline in tumor SUV of > 25% within tumor region, visible increase in extent of FDG tumor uptake (20% in longest dimension), or appearance of new ^18^F-FDG uptake in metastatic lesions	N.A	PD
**PERCIST** (64), 2009	Hottest single tumor lesion SUL of maximal 1.2cm diameter volume ROI in tumor (SUL peak)	Complete resolution of FDG uptake within measurable target lesion and disappearance of all other lesions to background blood pool levels.	> 30% relative decrease and > 0.8 absolute decrease in SULpeak of hottest lesion	Not meeting criteria for CMR, PMR, or PMD	> 30% relative increase and > 0.8 absolute increase in SULpeak of hottest lesion or unequivocal progression of ^18^F-FDG avid non-target lesion or appearance of new FDG avid	N.A	PD
**PECRIT** (67), 2017	All ^18^F-FDG–avid lesions at baseline as target lesions	Disappearance of all target lesions and non-target lesions; all lymph nodes < 10 mm short axis	≥ 30% decrease in sum of diameters of target lesions; non-target lesions may persist but not unequivocally progress	Neither sufficient tumor regression nor tumor growth to qualify for PMR or PMD percent change in SUL peak per PERCIST at 3–4 weeks. •SUL peak ≤ 15.5% → No clinical benefit •SUL peak > 15.5% → Clinical benefit	≥ 20% increase in sum of diameters of target lesions or unequivocal progression of non-target lesion or appearance of new lesion	N.A	PD
**PERCIMT** (68), 2018	Circumscribed sites of non-physiological ^18^F-FDG uptake greater than the background or liver activity	Complete resolution of all pre-existing FDG avid lesions. No new FDG avid lesions	Complete resolution of some pre-existing FDG avid lesions. No new FDG avid lesions	Neither PMD nor PMR/CMR	≥ 4 new lesions of less than 1 cm in functional diameter or ≥ 3 new lesions of more than 1.0 cm in functional diameter or ≥ 2 new lesions of more than 1.5 cm in functional diameter	N.A	Cut-off of four new lesion
**imPERCIST5** (69), 2019	Up to 5 focal, abnormally increased ^18^F-FDG uptake versus background regardless the presence of corresponding anatomic lesion on the CT scan.	Defined as the resolution of all malignant lesions and was nominally assigned as SULpeak of zero for quantitative analysis	If the sum of SULpeak decreased by at least 30%	Not meeting the definitions for CMR, PMR, or PMD	Increase of the sum of SULpeak of the 5 lesions by 30%	N.A	New lesions were included in the sum of SULpeak if they showed higher uptake than existing target lesions or if fewer than 5 target lesions were detected on the baseline scan.
**iPERCIST** (70), 2019	Similar to PERCIST	Similar to PERCIST	Similar to PERCIST	Not meeting criteria for PMD nor PMR/CMR	≥ 30% SULpeak increase, or new ^18^F-FDG-avid lesions (uPMD)	UPMD	Confirmation needed after 4–8 weeks (CPMD)

CMR, complete metabolic response; PMR, partial metabolic response; uPMR, unconfirmed PMR, SMD, stable metabolic disease; PMD, progressive metabolic disease; PD, progressive disease; SUV, standardized uptake value; SUL, SUV normalized by the lean body mass; EORTC, European Organisation For Research And Treatment Of Cancer; PERCIST, Positron Emission Tomography (PET) Response Criteria in Solid Tumors; PECRIT, PET/CT Criteria for Early Prediction of Response to Immune Checkpoint Inhibitor Therapy; PERCIMT, PET Response Evaluation Criteria for Immunotherapy; imPERCIST5 immunotherapy-modified PERCIST; iPERCIST, immune PERCIST. N.A., not applicable.

**Table 3:** Summary of metabolic findings in association with the metabolic criteria for the assessment of response to immunotherapy.

Context	Phenomena	Assessment	
**Tumor response**	Immunotherapy effect on tumor cells glycolytic activity.	**Metabolic criteria**	**PERCIMT** with PMD defined as the appearance of new lesions from 2 to 4 depending on lesion size (68).
			**imPERCIST5** with PMD defined as > 30% increase of the sum of SULpeak of the 5 target lesions defined on baseline PET/CT (69).
			**iPERCIST** with PMD defined as at least 30% increase of the sum of SULpeak of the 5 target lesions defined on baseline PET/CT (70).
		**Metabolic and morphologic criteria**	**PECRIT** with PMD defined as at least 20% increase in sum of diameters of target lesions or unequivocal progression of non-target lesion or appearance of new lesion (67).
**Pseudo-progression**	Avid inflammatory response with high glycolytic activity on ^18^F-FDG PET/CT.	**Unconfirmed PMD** on interim PET/CT according to **iPERCIST** (70), a new ^18^F-FDG PET/CT scan is required after 4-8 weeks to confirm or deny PMD.	
**Hyperprogression**	Important increase in tumor lesion metabolic volume.	**No robust criteria** though significantly higher **MTV and total tumor volume burden on follow up ^18^F-FDG PET/CT** in comparison to baseline scan might be useful (53).	
**Prognosis**	Association between tumor lesions metabolic activity or imaging phenotype and patients’ outcomes.	**Conventional PET measurements on baseline ^18^F-FDG PET/CT scan** (SUV, MTV and TLG) and association with overall survival (71).	
		**Specific radiotracers targeting PD-L1** for prediction of tumor response and assessment of tumor heterogeneity (72).	
		**Radiomics** for definition of imaging biomarkers based on texture features and clinico-biological factors (73).	

PMD, progressive metabolic disease; MTV, metabolic tumor volume; SUV, standardized uptake value; SUL, SUV normalized by the lean body mass; TLG, total lesion glycolysis; PET, ^18^F-FDG PET/CT, 2-deoxy-2-[^18^F]fluoro-D-glucose Positron Emission Tomography/Computer Tomography; PERCIMT, PET Response Evaluation Criteria for Immunotherapy; PERCIST, PET Response Criteria in Solid Tumors; imPERCIST5 immunotherapy-modified PERCIST; iPERCIST, immune PERCIST. PECRIT, PET/CT Criteria for Early Prediction of Response to Immune Checkpoint Inhibitor Therapy; PD-L1, programmed death-ligand 1.

##### 2.2.2.1 PECRIT

In a small population of 20 melanoma patients treated with anti-PD-1/PD-L1 combination therapy, functional and morphological parameters from early ^18^F-FDG-PET/CT were used and accurately predicted tumor response (67). Combining features of RECIST 1.1 and PERCIST, the PET/CT Criteria for Early Prediction of Response to Immune Checkpoint Inhibitor Therapy (PECRIT) were created and showed 100% sensitivity, 93%, specificity and 95% accuracy in predicting early tumor response (67). They could demonstrate that patients with changes in the FDG uptake which are classified as CR or PR according to RECIST 1.1 at the 3- or 4-weeks follow-up are more likely to maintain a durable response in the 4-month follow-up (67). According to PECRIT response assessment, patients with an increase of >15.5% in the SULpeak of the hottest lesion and SD (RECIST 1.1 definition) commonly present an improved tumor response (CR/PR) at 4 months or SD at 6 months (67) (**Table 2**).

##### 2.2.2.2 PERCIMT

The PET Response Evaluation Criteria for Immunotherapy (PERCIMT), which take into account the clinical relevance of the absolute number of new lesions during therapy, showed significantly higher sensitivity than the EORTC on early ^18^F-FDG-PET/CT in melanoma patients treated ipilimumab (68). They assessed patients’ best clinical response (median 21.4 month, range 6.3-41.9) and divided these patients subsequentially into a group with (SD, PR, and CR) and a group without (PD) clinical benefits. According to PERCIMT, all non-physiological foci with an uptake higher than the background or liver are defined as target lesions. Compared with EORTC, PERCIMT has a significantly higher performance in predicting the response to immunotherapy (93.6% sensitivity, 70% specificity, and 87.8% accuracy). PERCIMT was also validated on late ^18^F-FDG-PET/CT in a similar population treated with vemurafenib (selective BRAF inhibitor) and ipilimumab (74) (**Table 2**).

##### 2.2.2.3 imPERCIST5

The immunotherapy-modified PERCIST5 (imPERCIST5), an adaption of the PERCIST using the sum of SULpeak of up to 5 target lesions in a population of 60 metastatic melanoma patients treated with ipilimumab, were recently released (69). Progressive metabolic disease (PMD) was defined as 30% increase in this sum rather than the appearance of new lesions (69). Furthermore, according to their new definition of responders and non-responders, they found a significant difference in the OS at two years of 66% and 29%, respectively (*p*=0.003), indicating the potential of imPERCIST5 to predict prognosis (**Table 2**).

##### 2.2.2.4 iPERCIST

The frequency of indeterminate responses in patients undergoing immunotherapy led to the introduction of the immune PERCIST (iPERCIST) based on a dual-time-point evaluation and were proposed as nuclear imaging equivalent to iRECIST (19, 70). The iPERCIST is based on a retrospective study performed in non-small cell lung cancer patients treated with nivolumab who underwent 3 consecutive ^18^F-FDG-PET/CT scans: at baseline, after 4 cycles of treatment (after 8 weeks, first follow up) and after another 4 weeks (second follow up) (70). The definition of target lesions and most response categories was similar to PERCIST. However, the concept of unconfirmed PMD (uPMD), defined as PMD which is not confirmed at the first imaging follow-up, and confirmed PMD (cPMD), defined as the confirmation of PMD at the second imaging follow-up, were introduced (70). Comparable to iRECIST, the decision to pursue immunotherapy between first and second imaging follow up is evaluated according to patients’ clinical status and metabolic response (19) (**Table 2**).

Even if these criteria are promising, none of those were validated in prospective studies, which is precluding their implementation in current guidelines (22). Nevertheless, some recommendations were defined as a result of an immunotherapy symposium that took place at the European Association of Nuclear Medicine Annual Congress in 2017 (75). They suggested, that SUVpeak in particular may be useful to assess metabolic changes on ^18^F-FDG-PET/CT according PERCIST (75). In addition, both MTV and TLG values before and after treatment may also help to improve response monitoring (75). In patients with suspected pseudoprogression on interim PET/CT, particularly the number of new metabolic lesions could be predictive of PD, as it has been reported that the appearance of more than 4 lesions is associated with true progression (76).

Most recently, an international consortium of expert Societies established practice guidelines/procedure standards for the use of ^18^F-FDG-PET/CT in oncological patients undergoing immunotherapy, with special focus on response assessment in solid tumors (77). They recommend taking into account the appearance of new lesions when assessing the response,

-in terms of the number of anatomical sites and lesions,
-if other reasons could explain the appearance of the new lesions (irAEs or sarcoidosis, characterized by the growth of granulomas potentially in any part of your body, but usually affecting lungs, lymph nodes and the skin), and
-if the side is nodular (tumors drainage area, distribution suggestive of sarcoid-like lymphadenopathy)

In addition, it is recommended to perform MTV/TLG assessment at baseline and on subsequent studies. Finally, if there is any uncertainty between true progression versus pseudoprogression, especially at the first post-treatment follow-up, either a confirmatory ^18^F-FDG-PET/CT study at >4 weeks or biopsy should be performed (77).

##### 2.2.2.5 ^18^F-FDG-PET/CT and prognosis

Currently, there is no consensual cut-off value for changes in conventional ^18^F-FDG-PET/CT parameters such as for the SUVmax between baseline and re-staging PET/CT to define the best tumor response (67, 76). However, a prospective study done in patients with advanced non-small cell lung cancer found that the metabolic response on ^18^F-FDG-PET/CT after one month of nivolumab treatment was an independent prognostic factor showing significant difference in PFS between partial metabolic response (PMR) and non-PMR patients, suggesting the usefulness of semi-quantitative measurements to define tumor responses (78). Similarly, interim ^18^F-FDG-PET/CT 8 weeks after initiation of nivolumab treatment in patients with refractory or relapsed Hodgkin lymphoma could identify more patients with CR compared to CT alone (79). Moreover, both PET/CT and CT were predictive of OS (79).

Interestingly, while SUVmax has been reported to correlate with PD-L1 status and thus response to ICIs, it appears that SUVmax alone is a less of a robust imaging biomarker for survival prediction (80–82). In contrast, other ^18^F-FDG-PET/CT parameters such as SUVpeak, MTV, and TLG showed the ability in predicting outcomes in melanoma patients (66, 71). A retrospective study demonstrated that patients with higher total MTV and bone marrow-to-liver SUVmax ratio had a significantly shorter OS, whereas a low TLG was associated with the best overall response (71). In patients with mucosal and cutaneous melanoma treated with ICIs, the association between a spleen-to-liver-ratio >1.1 on baseline ^18^F-FDG-PET/CT and poor outcomes has been reported (83). Besides, there is evidence, that the amount of intratumoral necrosis according to a recently proposed ratio between metabolic-to-morphological lesion volumes on ^18^F-FDG-PET/CT might provide diagnostic clues for prognosis prediction in lung cancers patients (84). Indeed, lower ratios were associated with higher PD-L1 expression and better survival (84). Moreover, the combination of PET/CT-parameters and surrogate biomarkers might even better predict response and prognosis in ICI treated patients. The total MTV >75 cm^3^ and a derived neutrophil-to-lymphocyte ratio >3 were significantly associated with shorter OS, the latter was additionally associated with PFS (85). A composite biomarker consisting of a neutrophil-to-lymphocyte ratio <4.9 and TLG <149.5, the so called “immune-metabolic-prognostic index”, was defined (86). This index has shown to estimate the risk of disease progression and predict survival 8 weeks following ICI treatment initiation (86). Finally, another potential use of ^18^F-FDG-PET/CT in the follow-up of patients treated with ICI is to guide clinicians in the decision of eventual therapy discontinuation for safety reasons (87).

Regarding other PET tracers, in melanoma and lung cancer patients with brain metastasis, ^18^F-fluoro-ethyl-tyrosine (^18^F-FET)-PET/CT was able to distinguish with high accuracy (85%; *p*=0.003) between brain metastasis relapse versus treatment-related changes and showed that metabolic responders had significantly longer and stable follow-up (88). This is all the more interesting because the physiological ^18^F-FET uptake in the brain limits the response assessment of brain metastasis in ^18^F-FET-PET/CT.

### 2.3 Role of imaging in the peri-interventional management of intratumoral immunotherapy

HIT-IT is based on the simple principle of direct injection of immunotherapeutic agents into the tumor *via* percutaneously placed needles. This novel treatment approach is in rapid development and has shown promising results in recent phase III trials (38, 89). Especially, since these procedures are image-guided, the radiologist has a novel and important role in the peri-interventional management to ensure procedural safety, optimal response assessment and reproducible outcomes.

#### 2.3.1 Prioritization and baseline characterization of lesions for injection

Prioritizing tumor lesions for HIT-IT injections involves a complex set of components, including tumor characterization, tumor visibility and accessibility, as well as procedural safety (89). Both clinical experience and a comprehensive imaging assessment with CT and magnet resonance imaging are hereby required (16). Emerging or rapidly growing lesions distant to recently treated tumors are associated with rapidly proliferating neoplastic cells and are visible on these imaging modalities (89, 90). In addition, contrast-enhanced imaging allows for the detection of high vascularization as indicator of tumor activity (91). However, being cheap, easily accessible, ultrasound is most commonly used in clinical scenarios, especially for subcutaneous lesions. It should preferably be performed by the same operator during follow-up examinations.

The first consideration while selecting lesions for HIT-IT, is to ensure patient safety by reducing operational complexity and the potential risk for complications. The most important safety concern is probably the vascularization of the target lesion, as high vascularity may be associated with an increased risk of bleeding and accidental systemic administration of the drug (16)

Another consideration is the accessibility of the lesion for needle targeting. Depending on the operator’s experience, all tumors are potentially injectable. However, especially for lesions that are difficult to access, additional imaging or technical modalities may help to achieve optimal results. While superficial injections, e.g. of skin lesions and superficial lymph nodes, can be straightforward, deeper injections, such as of the liver, lungs, or deep lymph nodes, will undoubtedly require imaging. Moreover, additional techniques such as hydrodissection or carbodissection, may be used. A small needle is advanced under imaging guidance between the targeted lesion and the anatomical structure to be displaced. Once adequately positioned, saline or glucose solution (for “hydrodissection”), or carbon dioxide (for “carbodissection”), is administered through the needle in order to safely displace interpositioned structures and access the targeted lesion (92). Some central nervous and peritoneal injections may even require surgical interventions (93). The most common target regions are, therefore, subdermal soft tissues, muscles, and superficial lymphatic chains. Deep organs should be considered as second option. Liver lesions are still commonly targeted, as the liver is frequently affected by metastasis of different tumor origins.

Finally, the size of the lesion, the amount of viable tumor tissue, and necrosis, if any, must also be considered in the overall assessment. In clinical trials, injected tumor sites should be greater than 1 cm in diameter (>1.5 cm for lymph nodes) to ensure accurate and reproducible intratumoral drug delivery (89). Additionally, larger lesions are more likely to release higher amounts of tumor-associated antigens and elicit a broader adoptive immune response (93). However, injection of very large lesions (> 5cm) should be questioned or even avoided because they are frequently morphologically heterogeneous with central necrosis (often radiological visible), which complicates the homogeneous distribution of the immunotherapeutic agents and poses an increased risk of bleeding (89). Importantly, tumor sites with radiologic evidence of aggressiveness, such as local invasiveness, should be given higher priority for injection (16). In addition, better results can be observed after injection of tumors containing large amounts of viable tumor cells, often identified as metabolically active lesions on PET/CT, as new or expanding tumors distal to recent locoregional treatment on CT/MRI, or by elevated vascularity on contrast-enhanced CT/MRI (89). In contrast, necrotic or fibrotic tumors are often more immune tolerant and should not be prioritized for injections (89). By now, there is no clear evidence-based consensus of how many tumors to inject (89, 93). A summary of factors regarding lesion prioritization in the setting of HIT-IT injections is summarized in **Table 4**.

**Table 4:** Summary of factors for prioritizing lesions for injection.

Tumorcharacterization	Tumor	Tumoraccessibility	Tumormorphology	Safety	Multidisciplinary discussion
Clinical parameters	**Tumors:** >1cm and <5cm	**Superficial injections:** e.g. of the skin and superficial lymph nodes → no additional imaging needed	**Signs of aggressiveness:** e.g. local invasiveness	**Consider lesion vascularization:** e.g. risk of bleeding, accidental systemic administration	Lesion count for injections (single vs. multiple)?
Cross-sectional imaging: - CT - MRI - PET/CT	**Lymph nodes:** >1.5cm	**Deeper injections:** e.g. liver, lungs, and deep lymph nodes → CT/US → may require interventional techniques, e.g. hydrodissection, carbodissection	**Target viable tumor cells:** e.g. identified as metabolic active lesion (PET/CT), as new or enlarging tumors distant to any recent locoregional therapy or by high vascularization (CT/MRI)	**Liver:** avoid subcapsular lesions or lesions close to the bile tree	Several injections in the same anatomic region vs. different organs?
US		**Complex injections:** e.g. central nervous and peritoneal injections → may require surgical intervention		**Procedural complexity** and **operators experience**	

CT, computer tomography; MRI, magnetic resonance imaging; US, ultrasound; PET, positron emission tomography.

Treatment beyond disease progression can be performed depending on clinical parameters, especially in the case of dissociated response with a PD of not-injected lesions and PR/CR of injected lesions. In this situation, injections might be prioritized for progressive or new lesions.

#### 2.3.2 Capturing data during the procedure and HIT-IT follow-up

In the peri-interventional management, preferably CT-scans are used to calculate the drug dose and injection volume per lesion. Proper documentation of the lesions to be injected is thus essential. In addition, image guidance is critical for successful percutaneous needle access and positioning of the needle within the tumor lesion. It is important to appreciate, that at any time of intratumoral injection, the radiologist can acquire imaging data and perform primarily evaluations of injection status and treatment efficacy prior to the protocol assessment (89, 90). Moreover, non-target injections and complications such as bleeding can be anticipated early.

Reliable identification of injected lesions in the follow-up of each treatment cycle might be difficult. Indeed, there may be e.g. multiple lesions in the same organ or anatomic region that were injected. It is thus highly recommended to adequately document each case and to take screenshots of the tumor site at baseline imaging and immediately before each injection to provide information to identify injected lesions in the follow-up imaging assessment (89).

## 3 Current challenges, further directions, and potential imaging biomarkers

Immunotherapies, especially ICIs, have become part and parcel of cancer patients’ treatment. Hereby radiologists are encountering the problem of accurately and reproducibly assessing the specific tumor response patterns and various imaging criteria have been developed. In clinical trials, iRECIST is the most promising one (94). The assessment of response to cancers other than melanoma and lung cancer may cause further problems for accurate and reproducible response evaluation. Indeed, most data has been generated with these cancers, so further research with other cancer types is needed. Moreover, it is necessary for the radiologist to be aware about the clinical trial specifications and to evaluate the response criteria accordingly in order to avoid error and to ensure optimal response assessment.

Further issues arise with the increasing use of non-ICI immunotherapies (e.g., oncolytic viruses, cytokines, cancer vaccines, and adoptive cell transfer) and dual checkpoint inhibition, or combinations of immunotherapies (in particular ICIs) and conventional chemotherapy or locoregional treatments (e.g. ablation therapy or selective internal radiation therapy). This leads to the challenge of selecting the most accurate tumor response assessment criteria in a specific patient. For the assessment of dual checkpoint inhibition, multiple criteria are often combined. The iRECIST is particularly appropriate for this purpose, as it shares the same criteria for lesion selection and response assessment with the RECIST 1.1, except for the need for a confirmatory imaging follow-up at 4 to 8 weeks (19). This comparability allows for direct comparison of the criteria within clinical trials and facilitates communication between radiologists and oncologists in clinical scenarios (**Figure 9**). However, these combined criteria should always be used with caution as no clear consensus exists. Moreover, combination ICI-treatment triggers increased immune-related toxicity compared to ICI-monotherapy (55%-60% vs. 0.4%-41.2%) and often requires treatment discontinuation (11, 12, 95). In clinical practice, a major challenge is therefore to detect these toxicities as early as possible to ensure close patient monitoring and therapeutic management. Of particular importance in this context is the fact that 74% of these immune-related adverse events (95 confidence interval: 63-84%) are detectable on imaging modalities (^18^F-FDG-PET/CT: 83%, MRI: 83%, CT: 79%, and ultrasound: 70%) (96). Even more interestingly, they can be seen in 17% of the patients even before onset of clinical symptoms (12, 97, 98).

Especially in cases where morphological criteria are not conclusive, ^18^F-FDG-PET/CT has demonstrated its potential in assessing the tumor response following immunotherapy. In addition, there is evidence that ^18^F-FDG-PET/CT features can even be used to predict patient’s outcome (66, 71). Moreover, metabolic response criteria have been proposed and provide important diagnostic clues (21). However, the complexity of monitoring tumor response in ICI-treated patients prompted the development of novel radiotracers targeting e.g. CD8-positive T-cells such as with ^89^Zr-Df-IAB22M2C (^89^Zr-Df-Crefmirlimab) and ^18^F-arabinofuranosyl guanine (72). Especially PD-L1 tracers for PET/CT, which are currently available in clinical practice, showed a strong correlation with the PD-L1 status determined on immunohistochemistry (72). Moreover, these tracers were able to image the heterogeneity of PD-L1 expression on PET/CT between different patients and within tumor lesions in the same patient even more precisely than immunohistochemistry stained biopsy samples (72, 99–101). The histological PD-L1 expression status is clinically used for patient selection for PD-(L)1-combination treatments. However, the positive predictive value of PD-L1 in immunohistochemistry is low and limited PD-(L)1 therapy responses (45%) have been reported even in patients with 50% PD-L1 positive expression status (72). Importantly, using ^89^Zr-atezolizumab, a better correlation of tumor response to ICI therapy was found on PET/CT compared to immunohistochemistry assessment of the PD-L1 expression (101). Besides, ^89^Zr-durvulumab has been investigated in the PINCH trial (NCT03829007), a clinical and imaging prospective multicenter phase I-II study in patients with advanced head and neck squamous cell carcinoma treated with durvalumab (anti-PD-L1) (102). ^89^Zr-durvulumab’s safety could be shown, but its uptake did not correlate to durvalumab treatment response (102).

Big challenges remain in classical radiological image analysis such as the interobserver variance and its time-consuming nature. The use of machine learning, however, could improve current workflows in radiology, including standardization of image interpretation, the enhanced image quality, and creating databases for studies (103). Selecting the right patients and predicting treatment responses are still among the main problems of cancer immunotherapy in clinical practice. The use of machine learning based blood biomarkers, such as neutrophil-to-lymphocyte ratio and platelet-to-lymphocyte ratio, have already been shown promising results in for patient selection and predicting the treatment outcome in non-small cell lung cancer patients treated with nivolumab (104). There is, moreover, an increasing interest in radiomics models trained the clinical outcomes (105). Radiomics uses large numbers of features extracted with data characterization algorithms from medical imaging to define tumor patterns and features that are not visible to the human eye. The first study in this context used baseline and follow-up CT scans in non-small cell lung cancer and melanoma patients treated with anti-PD-1 to create a CT-derived radiomics biomarker to predict response to cancer immunotherapy (106). Their results showed that lesions with non-uniform morphological profiles, compact borders and heterogenous density patterns, were associated with response to immunotherapy. However, the performance of their model was good in individual lung cancer lesions (area under the curve 0.83, p< 0.001) but rather poor in melanoma lesions (area under the curve 0.64, p<0.05), which was explained by their higher number of pre-treatment exposures of these lesions. In addition, models have been developed, that extract peri- and intra-tumoral features from pre- and follow-up CTs of ICI treated patients and showed in both internal and external validation cohorts’ good performance in predicting therapy responses and OS (107). Regarding MRI imaging, an MRI-based deep learning algorithm capable of employing more layers of data proved to be useful in optimizing prostate cancer treatment and prognostication (108).

The field of radiomics may also offer additional information for the prediction of prognosis in patients treated with immunotherapy based on established molecular biomarkers (73, 105). A recent multi-center retrospective study of large populations of non-small cell lung cancer patients demonstrated the utility of a deep learning score based on radiomics features extracted from ^18^F-FDG-PET/CT to predict the PD-L1 expression status on immunohistochemistry (73). The further combination of this score with clinical features, including histology and Eastern Cooperative Oncology Group performance status, could accurately predict PFS and OS. An association between texture features extracted from ^18^F-FDG-PET/CT and PD-L1, PD-1, and CTLA-4 mRNA expression status in the tumor burden was reported in a small series of non-small cell lung cancer patients (109). Defining robust imaging biomarkers may be useful to non-invasively visualize tumor heterogeneity and target receptor expression to reduce the number of biopsies and ultimately improve clinical outcomes. Future investigations for a better understanding of immunotherapy response should include imaging, clinical and biological features, e.g. patient BMI or baseline plasma levels for PD-1 and PD-L1 which were previously reported to be associated with time-to-treatment-failure in a recent study involving Merkel cell carcinoma patients treated with avelumab (anti-PD-L1) (110).

The ^18^F-FDG-PET immunotherapy radiomics signature (iRADIOMICS), consisting of radiomics features, was able to predict response to immunotherapy in non-small cell lung cancer patients treated with pembrolizumab who underwent baseline and follow-up ^18^F-FDG-PET/CT (111). Interestingly, the radiomics multivariant analysis showed the best performance using baseline images (area under the curve 0.90) compared with baseline PD-L1 levels (area under the curve 0.60) and iRECIST at the 1- and 4-month follow-up (areas under the curves 0.79, 0.86, respectively). These findings are even more important, since the use of iRECIST requires a follow-up imaging session for the assessment, which delays the clinical decision making. iRADIOMICS in contrast has the potential to prognosticate ICI-treatment responses already on baseline imaging which may result in faster and more efficient patient management.

Moreover, there is preliminary data that showed that radiomics might help to predict immunotherapy-associated response patterns. Radiomics features were used from PET/CT to differentiate between true progression and pseudogrogression in melanoma patients (112). These features, especially in combination with blood parameters, may be promising biomarkers for early prediction of pseudoprogression. In addition, a recent study designed a classifier based on peritumoral (vascular) radiomics features extracted from pre-ICI treatment CT scans of non-small cell lung cancer patients, which showed to be indicative of hyperprogression (113). Nonetheless, further validation and testing of all these radiomics models across several cancer types is still needed to reliably predict treatment responses and specific response patterns in the setting of immunotherapy.

Taken together, these preliminary results show that radiomics and machine learning based imaging biomarkers might be useful for predicting response patterns, outcomes and prognosis of patients treated with immunotherapy, which might facilitate the application of cancer immunotherapy in clinical practice.

Immune-monitoring is another emerging field in the evaluation of immunotherapies to distinguish whether apparent tumor progression is a result of immune infiltration or actual progressive disease. Immune-monitoring allows specific targeting of an immune cell population or protein by conjugating a contrast agent with an antibody (114–116). This technique allows for *in situ* and real-time monitoring of T-cell tumor infiltration and functional status using molecular imaging tools without invasive histopathology (114–116). The focus of interest in many pre-clinical studies is imaging immune cell populations (e.g. CD2, CD3, CD4, CD7, CD8, CD11b, CD19, CD20), directly cells (e.g. dendritic cells, macrophages, chimeric antigen receptor T-cells, allogenic human T-cells, myeloid-derived suppressor cells), surface antibodies (e.g. PD1, PD-L1, CTLA-4, OX40), and cytokines (e.g. interferon gamma, transforming growth factor beta, interleukin 1 beta) with MRI, scintigraphy, PET/CT, or single photon emission CT (115). However, as far as we know, only a few imaging probes are currently under clinical investigation, and none have yet been approved by the FDA for clinical use. Moreover, the downstream physiological effects must be carefully considered when developing these imaging tracers for immunosurveillance, as antibodies could deplete the target cell population, trigger or inhibit receptor signaling, or neutralize the normal function of soluble proteins. The use of cytokines or other ligands as tracers may stimulate their respective signaling pathways. Thus, more data is needed.

Radiologists also have an emerging role in accompanying the peri-interventional management of HIT-IT, as imaging plays a critical role in patient and lesion selection during the procedure and in the follow-up. The simultaneous injection of radiopaque products with HIT-IT agents is being investigated in order to monitor the drug distribution and tag intraprocedurally the targeted tumor lesion with a fiducial markers for reliable injected lesion identification within each treatment cycle (e.g.: NCT03052205) (89, 90, 93). ^18^F-FDG-PET/CT has been suggested to be particularly helpful in the setting of HIT-IT in identifying active tumor(-components) and atypical tumor responses (89, 90). Moreover, a recently published preclinical study in a mouse model proposed its use for tracking the distribution of intratumoral injected drugs, intending to achieve maximal therapeutic effects (117).

Despite the growing use of HIT-IT, to date, there is also little consensus and no clear guidelines for the assessment in this area. In addition, there are many questions which have not been solved so far, such as: How many lesions should be injected in how many anatomic regions? Should always the same lesions be injected in the follow-up or when should lesions be re-prioritized for injections? Does simultaneous injection lead to boosting of the therapy effect? Does co-injection of different HIT-IT agents have beneficial effects? How long should the treatment be continued? Further investigations are thus needed to address these questions.

In conclusion, tumor response assessment on imaging following immunotherapy is challenging. A reproducible interpretation of morphological and metabolic imaging response criteria remains complicated in many clinical situations, especially if the radiologists and nuclear medicine doctors are unfamiliar with them. Therefore, there is still a need for easily applicable non-invasive criteria and biomarkers that allow optimal patient selection for the different immunotherapeutic approaches, toxicity screening, standardized response assessment, and outcome prediction in clinical trials.

## Author contributions

All authors listed have made a substantial, direct, and intellectual contribution to the work and approved it for publication.

## Funding

Open access funding was provided by the University of Lausanne.

## Conflict of interest

The authors declare that the research was conducted in the absence of any commercial or financial relationships that could be construed as a potential conflict of interest.

All figures included in the manuscript contain imaging performed at the Department of Diagnostic and Interventional Radiology at Lausanne University Hospital and have not been published in any previous publication.

## Publisher’s note

All claims expressed in this article are solely those of the authors and do not necessarily represent those of their affiliated organizations, or those of the publisher, the editors and the reviewers. Any product that may be evaluated in this article, or claim that may be made by its manufacturer, is not guaranteed or endorsed by the publisher.

## Glossary

**Table:**

CMR	complete metabolic response
cPMD	confirmed PMD
CR	complete response
CT	computer tomography
CTLA-4	cytotoxic T-lymphocyte-associated protein-4
EORTC	European Organisation for Research and Treatment of Cancer
PET	positron emission tomography
HIT-IT	human intratumoral immunotherapy
ICI	immune checkpoint inhibitor
icPD	confirmed PD
iDR	immune-dissociated response
imPERCIST5	immunotherapy-modified PERCIST5
imRECIST	immune-modified RECIST
iPERCIST	immune PERCIST
irAE	immunotherapy-related adverse events
iRECIST	immune RECIST
irRC	immune-related response criteria
irRECIST	immune-related RECIST
itRECIST	intra-tumoral RECIST
iuPD	unconfirmed progressive disease
LAG-3	anti-Lymphocyte Activation Gene-3
mAb	monoclonal antibody
MTV	metabolic tumor volume
OS	overall survival
PD	progressive disease
PD-1	programmed cell death protein-1
PD-L1	programmed death-ligand 1
PERCIMT	PET Response Evaluation Criteria for Immunotherapy
PERCIST	PET Response Criteria in Solid Tumors
PECRIT	PET/CT Criteria for Early Prediction of Response to Immune Checkpoint Inhibitor Therapy
PFS	progression free survival
PMD	progressive metabolic disease
PMR	partial metabolic response
PR	partial response
RECIST 1.1	Response Evaluation Criteria in Solid Tumors version 1.1
SD	stable disease
SMD	stable metabolic disease
SUL	SUV normalized by the lean body mass
SUV	standardized uptake value
T-VEC	Talimogene laherparepvec
TGR	tumor growth rate
TLG	total lesion glycolysis
TME	tumor microenvironment
uPMD	unconfirmed PMD
^18^F-FDG-PET/CT	2-deoxy-2-[18F]fluoro-D-glucose PET/CT
^18^F-FDG	2-deoxy-2-[^18^F]fluoro-D-glucose
^18^F-FET	^18^F-fluoro-ethyl-tyrosine
