# Erratum: Tumor response assessment on imaging following immunotherapy

**DOI:** 10.3389/fonc.2023.1175321

**Published:** 2023-03-07

**Authors:** Frontiers Production Office

**Affiliations:** Frontiers Media SA, Lausanne, Switzerland

**Keywords:** tumor response, immunotherapy, immune checkpoint inhibitor, pseudoprogression, iRECIST, imRECIST, PERCIMT, iPERCIST

An omission to the funding section of the original article was made in error. The following sentence has been added: “Open access funding was provided by the University of Lausanne”.

The original version of this article has been updated.

